# MicroRNA-profiling of miR-371~373- and miR-302/367-clusters in serum and cerebrospinal fluid identify patients with intracranial germ cell tumors

**DOI:** 10.1007/s00432-022-03915-4

**Published:** 2022-02-16

**Authors:** Stefan Schönberger, Mahsa Mir Mohseni, Jörg Ellinger, Giao Vu Quynh Tran, Martina Becker, Alexander Claviez, Carl-Friedrich Classen, Barbara Hermes, Pablo Hernáiz Driever, Norbert Jorch, Melchior Lauten, Marcus Mehlitz, Niklas Schäfer, Johanna Scheer-Preiss, Dominik T. Schneider, Anja Troeger, Gabriele Calaminus, Dagmar Dilloo

**Affiliations:** 1 Department of Pediatric Hematology and Oncology, University Hospital Bonn, Rheinische Friedrich-Wilhelms-Universität Bonn, Bonn, Germany; 2 Department of Pediatric Hematology and Oncology, University Hospital Essen, University of Duisburg-Essen, Hufelandstrasse 55, 45147 Essen, Germany; 3 Department of Urology and Center of Integrated Oncology (CIO), University Hospital Bonn, Bonn, Germany; 4 Department of Pediatric Hematology and Oncology, Goethe University Frankfurt, Frankfurt, Germany; 5 Department of Pediatrics, Pediatric Hematology and Oncology, Medical University of Schleswig-Holstein, Campus Kiel, Kiel, Germany; 6 University Children’s and Adolescents’ Hospital, Rostock University Medical Center, Rostock, Germany; 7 Kreiskliniken Reutlingen, Medizinische Klinik I, Reutlingen, Germany; 8 Department of Pediatric Oncology and Hematology, Charité-Universitätsmedizin Berlin, corporate member of Freie Universität Berlin, Humboldt-Universität zu Berlin and Berlin Institute of Health, Berlin, Germany; 9 Department of Pediatric Hematology and Oncology, Hospital of Bielefeld, Bielefeld, Germany; 10 Department of Pediatric and Adolescent Medicine, Pediatric Hematology and Oncology, University Hospital Schleswig-Holstein, Lübeck, Germany; 11 Department of Neurosurgery, Krankenhaus der Barmherzigen Brüder Trier, Trier, Germany; 12 Division of Clinical Neurooncology, Department of Neurology and Center of Integrated Oncology (CIO), University of Bonn, Bonn, Germany; 13 Department of Pediatric and Adolescent Medicine, Braunschweig Municipal Hospital, Brunswick, Germany; 14 Clinic of Pediatrics, Dortmund Municipal Hospital, Dortmund, Germany; 15 Department of Pediatric Hematology, Oncology and Stem Cell Transplantation, University Hospital Regensburg, Regensburg, Germany

**Keywords:** Intracranial germ cell tumors, Liquid biopsy, miR-371, miR-372, miR-367, miR-302d

## Abstract

**Purpose:**

Intracranial germ cell tumors (iGCT) comprise germinoma and non-germinoma. Their diagnosis predominantly relies on biopsy as only one-fifth of patients present with elevated biomarkers (AFP/ß-HCG) in serum or cerebrospinal fluid (CSF). MicroRNAs (miR/miRNA) have emerged as non-invasive biomarkers in extracranial GCT and may potentially facilitate non-invasive diagnosis in iGCT.

**Methods:**

We analyzed eight miRNAs in serum and CSF from the miR-371~373- and miR-302/367-clusters and four miRNAs differentially expressed in iGCT tissue (miR-142-5p/miR-146a-5p/miR-335-5p/miR-654-3p) from eight iGCT patients (age 10–33 years) and 12 control subjects by pre-amplified RT-qPCR. MiR-30b-5p (serum) and miR-204-5p (CSF) acted as reference genes. Δ*C*_t_-values were expressed as $2^{{ - \Delta \Delta C_{{\text{t}}} }}$ after standardization against controls.

**Results:**

Between iGCT and control patients’ serum Δ*C*_t_-values of miR-371a-3p (*p* = 0.0159), miR-372-3p (*p*= 0.0095, miR-367 (*p* = 0.0190), miR-302a (*p* = 0.0381) and miR-302d-3p (*p* = 0.0159) differed significantly. Discriminatory pattern in CSF was similar to serum as miR-371a (*p* = 0.0286), miR-372-3p (*p* = 0.0028), miR-367-3p (*p* = 0.0167) and miR-302d-3p (*p* = 0.0061) distinguished between patients and controls. Abundant $2^{{ - \Delta \Delta C_{{\text{t}}} }}$ levels of each of these miRNAs were found across all serum and CSF samples including biomarker-negative patients.

**Conclusion:**

With the largest data set so far, we underline the suitability of miR-371a, miR-372, miR-367 and miR-302d in serum and CSF for diagnosis of iGCT, particularly in biomarker-negative germinoma. Diagnosis of iGCT by miRNA analysis is a feasible and valid approach, particularly as serum can be readily obtained by a less invasive procedure. MiRNA analysis may discriminate iGCT from other tumors with similar radiological findings and may allow to monitor response to therapy as well as early relapse during follow-up.

## Introduction

Intracranial germ cell tumors (iGCT) constitute a heterogeneous group of brain tumors affecting children, adolescents, and young adults. Histologically, they comprise pure germinoma (GER) and non-germinomatous tumors (NGGCT), such as embryonal carcinoma (EC), choriocarcinoma (CHC), yolk sac tumor (YST) or teratoma (Murray et al. 2014). Localized in brain midline structures, especially in the pineal and suprasellar regions, they may present as bifocal iGCT at both sites simultaneously (Calaminus et al. 1994).

One-fifth of iGCT are detectable by elevated tumor markers in serum or cerebrospinal fluid (CSF) classifying these tumors as marker-positive iGCT (Calaminus et al. 1997). In all other iGCT diagnosis relies on invasive biopsy, which although nowadays an overall safe procedure still carries a risk (Schulz et al. 2021). Moreover, biopsies may not represent the entire tumor as the histologic composition of iGCT may be heterogeneous (Takami et al. 2019). Platin-based chemotherapy, surgery of residual lesions, and adjuvant radiotherapy are the treatment modalities of choice in iGCT in Europe. Yet, 10% of GER and 30% of NGGCT relapse within 5 years after intense multimodality therapy (Calaminus et al. 2017, 2013). As discrimination from other intracranial tumors and early detection are essential for immediate treatment initiation in iGCT, less invasive methods are warranted that facilitate reliable diagnosis.

MicroRNAs (miR/miRNA) are small non-coding RNAs that have emerged as non-invasive biomarkers. They are involved in the regulation of gene expression and are frequently dysregulated in cancers. Among these, the miR-371~373 and miR-302/367-clusters are overexpressed in malignant extracranial GCT regardless of the histologic subtype, tumor site, or patient age (Palmer et al. 2010; Gillis et al. 2007). We and others have previously analyzed the miR-371~373-cluster and miR-367-3p in serum of adults suffering from testicular cancer and were able to discriminate GCT patients from healthy subjects with high sensitivity (85–98%) and specificity (94–99%) (Syring et al. 2015; Gillis et al. 2013; Dieckmann et al. 2019). Testicular and intracranial GCT showed identical histologic patterns and similar genomic profiles (Takami et al. 2019; Schneider et al. 2006; Ichimura et al. 2016). Therefore, analysis of the GCT-specific miR-371~373 and miR-302/367-clusters in serum and/or CSF of iGCT patients seems a promising approach that is underlined by a pioneering publication on two iGCT patients (Murray et al. 2016).

In a global miRNA expression analysis of 12 tumor specimens from primary pediatric iGCT, GER-specific upregulation of miR-142-5p and miR-146a-5p was documented in contrast to overexpression of miR-335-5p and miR-654-3p in NGGCT (Wang et al. 2010). Yet, it is still unknown whether detection of these miRNAs is discriminatory in serum of iGCT patients versus healthy controls.

Here we present a comprehensive miRNA analysis in a case series of iGCT patients exploring 12 different microRNAs (miR-371a-3p, miR-372-3p, miR-373-3p, miR-302a-3p, miR-302b-3p, miR-302c-3p, miR-302d-3p, miR-367-3p, miR-142-5p, miR-146a-5p, miR-335-5p and miR-654-3p) in both serum and CSF at time of diagnosis.

## Patients and methods

### Patient enrollment

The study was approved by the local ethics committee of the medical faculty of the Rheinische Friedrich-Wilhelms-Universität of Bonn, Germany (No. 048/18). Adult patients or parents of minors provided full informed consent. Serum and/or CSF of eight iGCT patients was collected at diagnosis for miRNA analysis in eight different oncology centers in Germany between September 2018 and January 2020. From these eight patients, serum (*n* = 6) and/or CSF (*n* = 4) was obtained prior to treatment. In two iGCT patients, both serum and CSF was available at diagnosis. Diagnosis of NGGCT (*n* = 3) was based on elevated α-fetoprotein (AFP, > 25 ng/ml) and/or ß-human chorionic gonadotropin (HCG, > 50 IU/l) in serum and/or CSF. In marker-negative cases (*n* = 5) biopsy was performed and revealed pure GER. Mean patient age was 17.5 years (range 10.3–33.2). Patient characteristics are summarized in Table 1. Serum from healthy donors (*n* = 3) and one leukemia patient as well as CSF from children suffering from other malignant diseases (acute lymphoblastic leukemia, *n* = 5; other malignant brain tumors, *n* = 3) and from one case of pituitary inflammation was included as controls.

**Table 1:** Clinical data of eight patients with intracranial germ cell tumors (iGCT)

Pat.-ID	Age at diagnosis (years.months)	Type of iGCT	Histology	Available material for miRNA analysis	Initial serum AFP (IU/ml)	Initial CSF AFP (IU/ml)	Initial serum ß-HCG (IU/l)	Initial CSF ß-HCG (IU/l)
NGGCT#1	10.10	Non-germinoma	Biopsy not done	Serum, CSF	**93.0**	**16.1**	< 1.0	< 1.0
NGGCT#2	18.7	Non-germinoma	GER, CHC	Serum	2.2	< 1.0	**55.0**	**242.0**
NGGCT#3	14.11	Non-germinoma	Biopsy not done	Serum	2.2	< 1.0	**49.3**	**165.0**
GER#1	33.2	Germinoma	GER	Serum, CSF	< 1.0	< 1.0	< 1.0	< 1.0
GER#2	16.11	Bifocal germinoma	GER	Serum	< 1.0	< 1.0	< 1.0	< 1.0
GER#3	18.3	Germinoma	GER	Serum	< 1.0	< 1.0	< 1.0	< 1.0
GER#4	10.3	Bifocal germinoma	GER	CSF	< 1.0	< 1.0	< 1.0	< 1.0
GER#5	19.0	Metastatic germinoma	GER	CSF	< 1.0	< 1.0	< 1.0	< 1.0

Pat.-ID	Localization	Comorbidities at diagnosis
NGGCT#1	Pineal region	–
NGGCT#2	Pineal region	Impaired vision with diplopic images
NGGCT#3	Caudal of the left-sided ventricle	Diabetes insipidus
GER#1	Sellar region,	Aneurysma at the end of *A. basilaris*
GER#2	Pineal region, thickening of pituitary style	Diabetes insipidus, cerebral right-sided hemiplegia
GER#3	Pineal region	Diabetes insipidus, impaired vision with diplopic images
GER#4	Sellar and pineal region, thickening of pituitary style	Diabetes insipidus, hashimoto thyreoiditis, growth retardation
GER#5	Pineal region with metastatic spread	–

Values in bold type are above normal range

*NGGCT* non-germinomatous germ cell tumor, *GER* germinoma, *CHC* choriocarcinoma, *CSF* cerebrospinal fluid, *AFP* alpha-Fetoprotein, *ß-HCG* ß-human chorionic gonadotropin

### MicroRNA analysis

RNA isolation and microRNA quantification was performed based on a published protocol, which had already been implemented in miRNA analysis in GCT (Murray et al. 2016; Bell et al. 2017) and adopted in this study to achieve higher miRNA concentrations. Briefly, prior to RNA extraction MS2 carrier RNA (Roche, Welwyn Garden City, USA) was added to serum and CSF samples to increase RNA yield. To enhance RNA quantity for further microRNA analysis, RNA isolation was performed twice per sample and eluted in 20 µl each. Non-human spike-in cel-miR-39-3p was used to control for RNA extraction efficiency. Two samples presented an increased Δ*C*t^miR23a-miR-451^ between 9 and 11 indicating some degree of hemolysis. We proceeded to further analyze these two cases as miR-30b-5p was in the range of the other patient and control samples. TaqMan^®^ microRNA reverse transcription kit (Applied Biosystems, USA) was applied for multiplex reverse transcription of 5 µl RNA followed by a multiplex pre-amplification step with 12.5 µl cDNA using the TaqMan^®^ PreAmp Master Mix (Applied Biosystems, USA). Afterwards, the final product of 50 µl was diluted 1:1 (50 µl + 50 µl) with RNAse-free water. TaqMan^®^ Fast Advanced Master mix (Applied Biosystems, USA) was employed for the final singleplex qPCR of GCT-specific microRNAs being performed in duplicates and by adding 9 µl of pre-amplified cDNA to a final reaction volume of 20 µl per sample. Only samples yielding a maximum fluorescence value of at least 30 and exhibiting sigmoid-like shaped curves were included in the analysis. The mean of *C*_t_ values was normalized against the reference genes miR-30b-5p for serum and CSF as well as miR-204-5p for CSF (Δ*C*_t_). In addition, patient Δ*C*_t_ values were standardized against Δ*C*_t_ values of control samples and expressed as $2^{{ - \Delta \Delta C_{{\text{t}}} }}$. In this manuscript miRNAs targeting the 5′-untranslated region are labeled with “-5p” (miR-142-5p, miR-146a-5p, miR-335-5p, miR-30b-5p, miR-204-5p), miRNAs without additional notation bind to the 3′-untranslated region.

### Statistical analysis

The Mann–Whitney *U* Test was used to determine differences between two groups, e.g., iGCT patients and controls. *p* values < 0.05 were considered statistically significant.

## Results

### Evaluation of reference genes miR-30b-5p and miR-204-5p

**Fig. 1:**
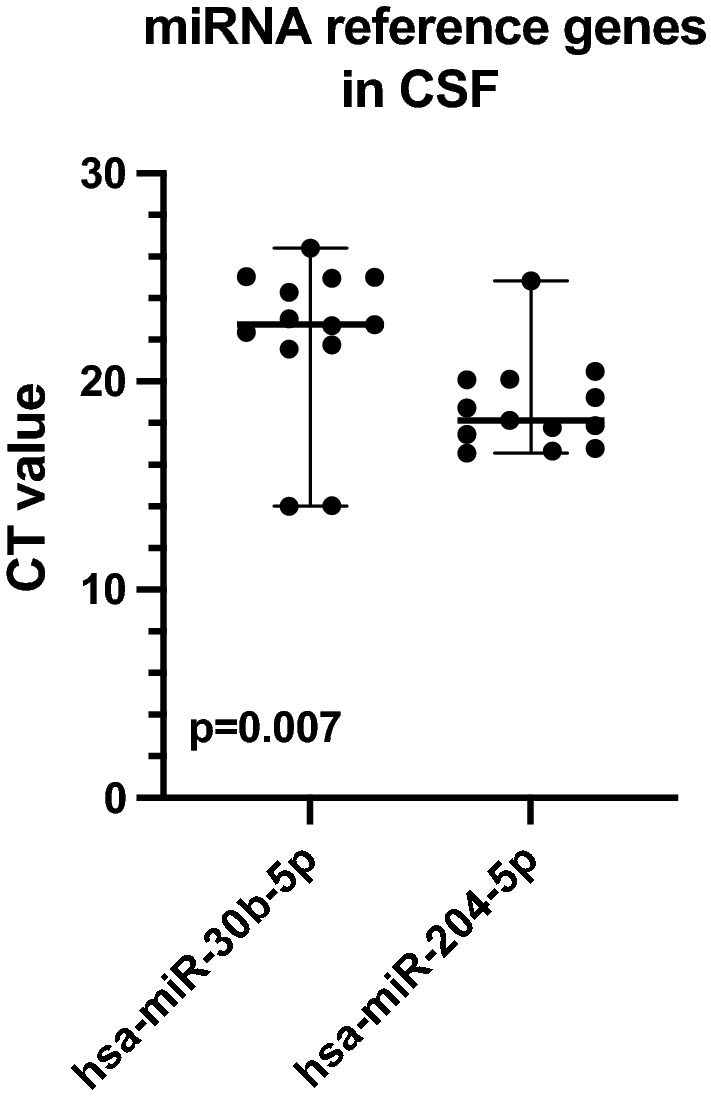
Comparison of miRNA reference gene expression of miR-30b-5p and miR-204-5p in CSF samples*.* *C*_t_ values of miRNA reference genes miR-30b-5p and miR-204-5p of initial patient (*n* = 4) and control (*n* = 9) CSF samples are presented as median and range

We compared *C*_t_ values of miR-30b-5p (serum + CSF) and miR-204-5p (CSF) for evaluation of reference gene expression (Fig. 1). Across all initial serum samples in patients (*n* = 6) and controls (*n* = 4) the median of miR-30b-5p *C*_t_ values was 15.48 (range 12.68–21.41) indicating robust reference gene expression in serum. In CSF, the median miR-30b-5p *C*_t_ value was 22.74 (range 14.02–26.41) of patients (*n* = 4) and controls (*n* = 9), while *C*_t_ values of the alternative reference gene miR-204-5p were significantly lower with a median of 18.14 and a narrower range (16.55–24.83; *p* = 0.007). Consequently, miR-204-5p was selected for normalization of target CSF miRNA.

### Serum levels of miR-371a, miR-372, miR-367, miR-302a and miR-302d identify patients with intracranial germ cell tumors

**Fig. 2:**
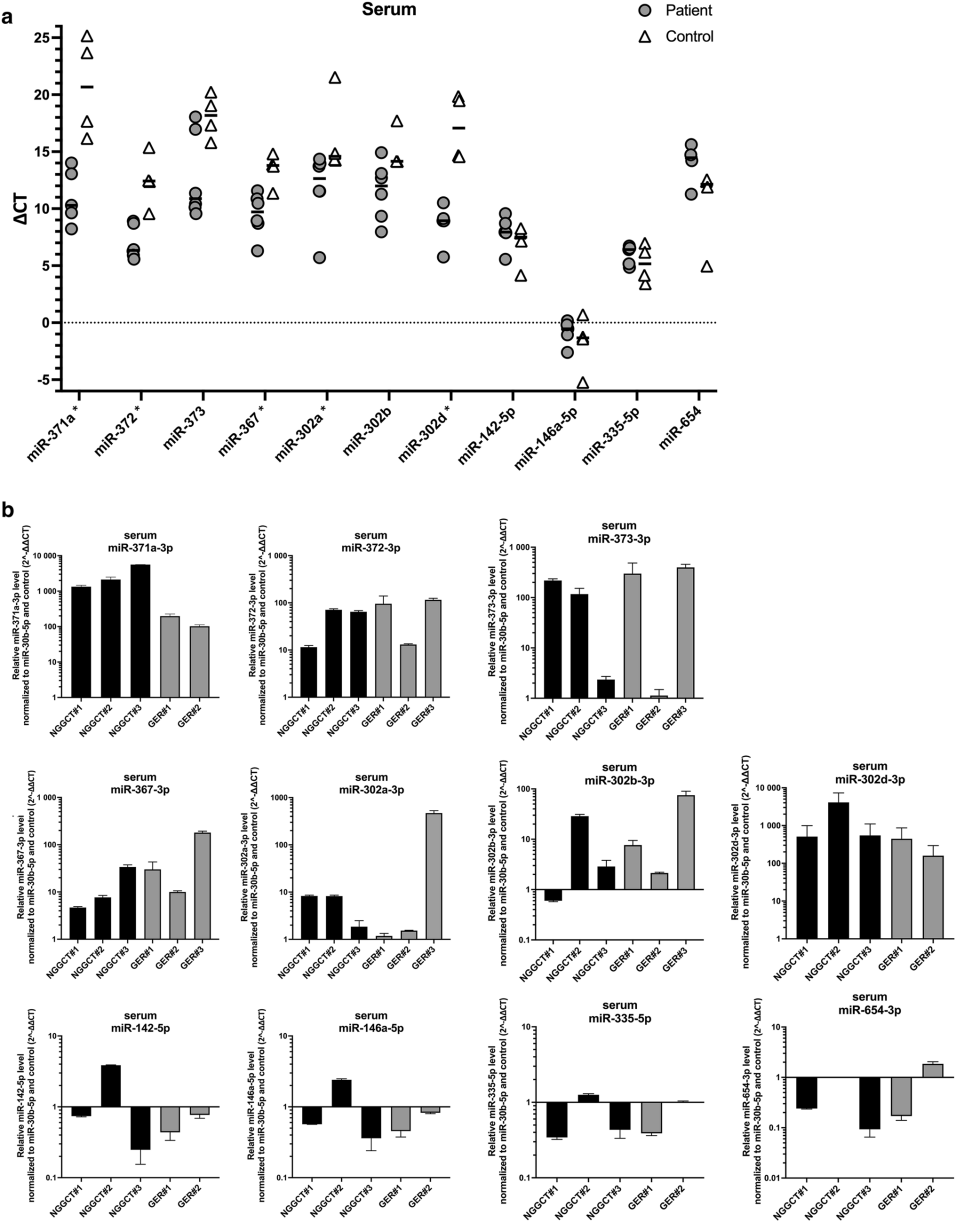
Analysis of different serum miRNAs in patients diagnosed with iGCT and controls. **a** Mean of CT duplicates was normalized against the mean of CT values of the reference gene miR-30b-5p and expressed as a grey point (patient sample) or a clear triangle (control sample). Horizontal black lines indicate the median values of patient (*n* = 5–6) or control samples (*n* = 3–4). MiRNAs being statistically different overexpressed in iGCT patients compared to controls are marked with an asterisk. **b** Mean of CT duplicates was normalized against both miR-30b-5p and normalized CT values of controls and expressed as $2^{{ - \Delta \Delta C_{{\text{t}}} }}$ level for every patient suffering from non-germinomatous (black bars) or germinomatous (grey bars) iGCT

In serum, *C*_t_ values were normalized to the reference gene miR-30b-5p (Δ*C*_t_). Among the 12 tested microRNAs, miR-371a (*p* = 0.0159), miR-372 (*p* = 0.0095), miR-367 (*p* = 0.0190), miR-302a (*p* = 0.0381) as well as miR-302d (*p* = 0.0159) differed significantly between iGCT patients and control subjects (Table 2, Fig. 2a). In contrast, Δ*C*_t_ values of miR-373 (*p* = 0.0667) and miR-302b (*p* = 0.0952) known to be overexpressed in serum of patients with extracranial GCT failed to discriminate between patients suffering from iGCT and controls. MiR-302c-qPCR repetitively failed to present sigmoidal curves but exhibited rather linear slopes with a fluorescence level < 25 in both patients and controls. Consequently, analysis of miR-302c was entirely excluded from further analysis. MiR-142-5p, miR-146a-5p, miR-335-5p, and miR-654 differentially expressed in tissue of iGCT patients showed appropriate amplification curves yet without any significant difference between serum of patients and controls.

**Table 2:** Median Δ*C*_t_ and range of 11 different microRNAs in serum of controls and patients suffering from intracranial germ cell tumors (iGCT)

Serum	Controls		Patients suffering from iGCT		*p*
miR-	Median Δ*C*_t_	Minimum–maximum	Median Δ*C*_t_	Minimum–maximum	
371a	20.68	16.16–25.17	10.30	8.22–14.01	**0.0159**
372	12.43	9.56–15.36	6.34	5.57–8.90	**0.0095**
373	18.19	15.80–20.23	10.89	9.56–18.04	0.0667
367	13.80	11.35–14.81	9.71	6.30–11.57	**0.0190**
302a	14.59	14.25–21.52	12.64	5.71–14.35	**0.0381**
302b	14.16	14.15–17.70	11.99	7.97–14.92	0.0952
302d	17.08	14.56–19.85	8.95	5.76–10.52	**0.0159**
142-5p	7.51	4.17–8.27	7.94	5.56–9.57	0.2857
146a-5p	− 1.34	− 5.23–0.70	− 0.53	− 2.61–0.17	0.5556
335-5p	5.17	3.42–6.96	6.41	4.85–6.72	0.5556
654	12.15	4.96–12.55	14.47	11.27–15.62	0.2000

*p* values < 0.05 were considered statistically significant

For further analysis, patient Δ*C*_t_ values were standardized to the median Δ*C*_t_ of controls and expressed as relative miRNA level ($2^{{ - \Delta \Delta C_{{\text{t}}} }}$; Fig. 2b). Abundant relative levels of miR-371a and miR-302d were found across all patient samples. The median relative level was 1333 for miR-371a (range: 102–5615) and 283 for miR-302d (94–2548). In addition, miR-372 and miR-367 displayed pronounced expression with median $2^{{ - \Delta \Delta C_{{\text{t}}} }}$ level of 68 (11–116) and 19 (5–180), respectively. In miR-373 an increase of relative miRNA expression was observed in 4/6 iGCT patients with a median of 243 (115–395). With exception of NGGCT#1, miR-302b was markedly expressed in 5/6 patients with a median $2^{{ - \Delta \Delta C_{{\text{t}}} }}$ level of 7 (2–73). Relative expression levels > 2 were also detected in 3/6 patients for miR-302a.

In keeping with results from testicular GCT on the 371~373-- and 302/367-clusters, $2^{{ - \Delta \Delta C_{{\text{t}}} }}$ levels of each serum miRNA did not significantly differ between patients with non-germinomatous and germinomatous iGCT (each *p* > 0.5). Of note, in two biomarker-negative patients (GER#1, GER#2) without elevated AFP and/or ß-HCG in serum and/or CSF at diagnosis, miR-371a and miR-302d were markedly overexpressed and thus informative. In the other biomarker-negative patient GER#3, miR-371a and miR-302d yielded only *C*_t_ values of low fluorescence intensity. All other $2^{{ - \Delta \Delta C_{{\text{t}}} }}$ levels of miRNAs in this patient from the 371~373- and 302/367-clusters were strikingly elevated ranging from 75–471 and thus allowing diagnosis of iGCT.

While expression of miR-142-5p and miR-146a-5p was enhanced in one patient (NGGCT#2) suffering from a NGGCT comprising CHC and GER histology, no patient presented elevated $2^{{ - \Delta \Delta C_{{\text{t}}} }}$ levels of miR-335 and miR-654. As miR-142-5p, miR-146a-5p, miR-335 and miR-654 harbored no additional value on discrimination of iGCT from controls as already shown in Fig. 1, further miRNA analysis in CSF focused on the 371~373- and 302/367-clusters.

### In CSF miR-371a, miR-372, miR-367 and miR-302d also allowed for discrimination of patients with intracranial germ cell tumors from controls

**Fig. 3:**
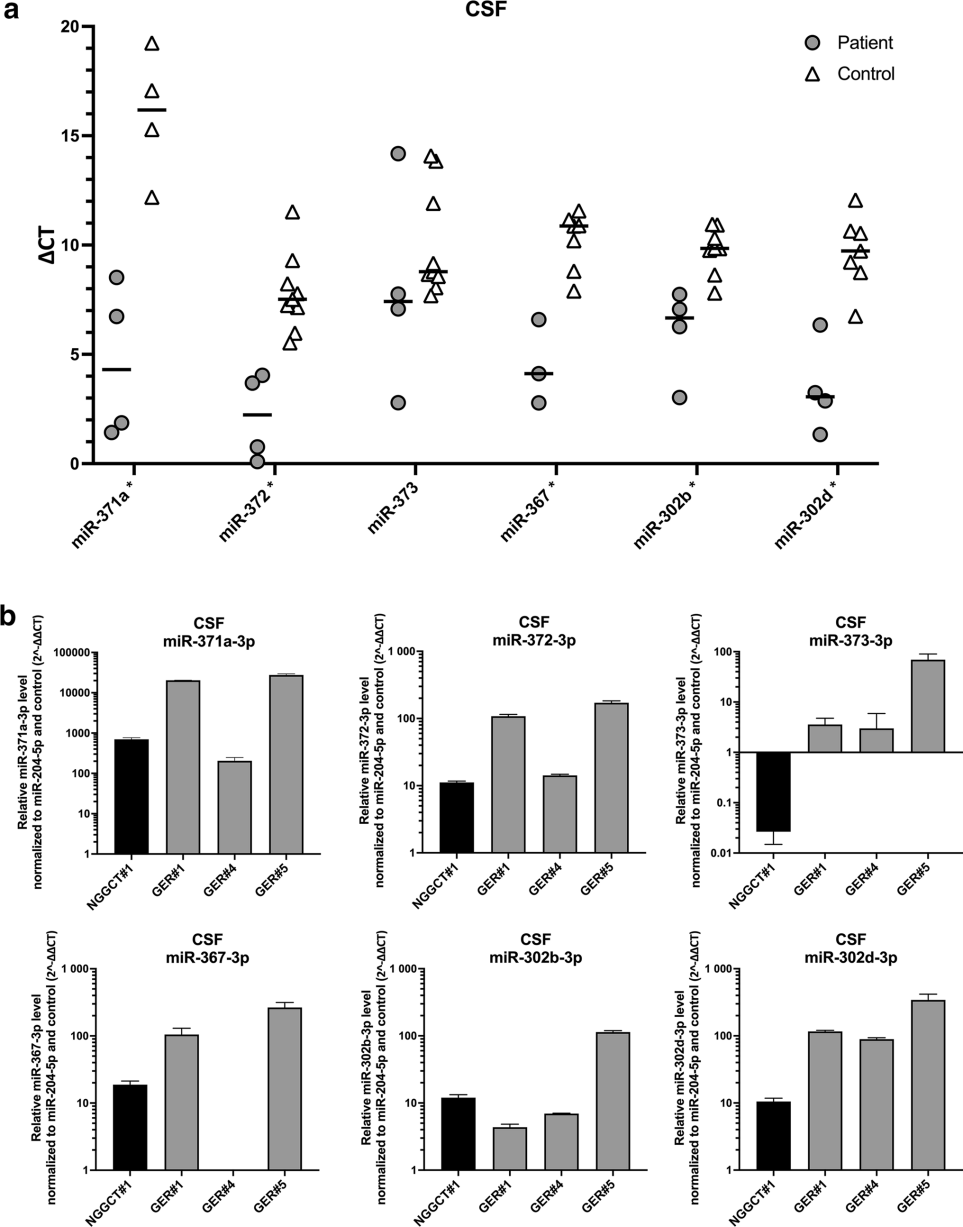
Analysis of different CSF miRNAs in patients diagnosed with iGCT and controls. **a** Mean of *C*_t_ duplicates was normalized against the mean of *C*_t_ values of the reference gene miR-204-5p and expressed as a grey point (patient sample) or a clear triangle (control sample). Horizontal black lines indicate the median values of patient (*n* = 3–4) or control samples (*n* = 4–9). MiRNAs being statistically different overexpressed in iGCT patients compared to controls are marked with an asterisk. **b** Mean of *C*_t_ duplicates was normalized against both miR-204-5p and normalized *C*_t_ values of controls and expressed as $2^{{ - \Delta \Delta C_{{\text{t}}} }}$ level for every patient suffering from non-germinomatous (black bars) or germinomatous (grey bars) iGCT

In CSF, the housekeeper miR-204-5p and most of the analyzed miRNAs presented with adequate maximum fluorescence levels. Only in miR-371a analysis of 5/9 control samples maximum fluorescence was exceedingly low and although considered negative had to be excluded from the analysis as no valid CT value could be attributed. The remaining four control and four patient samples were evaluable and Δ*C*_t_ values of miR-371a were discriminatory (*p* = 0.0286). Discriminatory pattern of the other miRNAs in CSF was similar to serum as miR-372 (*p* = 0.0028), miR-367 (*p* = 0.0167) and miR-302d (*p* = 0.0061) in iGCT patients differed from controls. In contrast to serum, expression of miR-302b in CSF also separated patients from controls (*p* = 0.0040), while miR-373 did not differentiate between these groups (*p* = 0.2601; Table 3, Fig. 3a). In CSF in spite of adequate maximum fluorescence levels, miR-302c and miR-302a did not retrieve sigmoidal-like curves for both patients and controls with the established primer set resulting in the exclusion of these miRNAs from analysis.

**Table 3:** Median Δ*C*_t_ and range of 7 different microRNAs in cerebrospinal fluid of controls and patients suffering from intracranial germ cell tumors (iGCT)

CSF	Controls		Patients suffering from iGCT		*p*
miR-	Median Δ*C*_t_	Minimum–maximum	Median Δ*C*_t_	Minimum–maximum	
371a	16.18	12.19–19.24	4.30	1.43–8.52	**0.0286**
372	7.52	5.53–11.52	2.23	0.10–4.04	**0.0028**
373	8.78	7.69–14.08	7.42	2.79–14.18	0.2601
367	10.87	7.90–11.57	4.12	2.78–6.59	**0.0167**
302a	12.19	7.35–16.38	5.86	5.06–6.65	0.1333
302b	9.85	7.81–10.95	6.67	3.03–7.74	**0.0040**
302d	9.73	6.75–12.06	3.06	1.33–6.34	**0.0061**

*p* values < 0.05 were considered statistically significant

Upon calculation of CSF miRNA levels in iGCT patients relative to controls employing the $2^{{ - \Delta \Delta C_{{\text{t}}} }}$ method (Fig. 3b), $2^{{ - \Delta \Delta C_{{\text{t}}} }}$ levels were highest in miR-371a across all patient samples with a median of 10,524 (range 203–27,600). Furthermore, every patient displayed very high relative levels of miR-372 (median, range: 61, 11–171), miR-367 (108, 19–273), miR-302b (9, 4–113) and miR-302d (103, 10–339). All germinoma patients but not the patient suffering from NGGCT exhibited overexpression of miR-373 with median relative levels of 3 (2–64). Of note, the three patients negative for the biomarkers AFP and ß-HCG from whom CSF was obtained at diagnosis (GER#1, GER#4, GER#5) exhibited pronounced expression of miR-372 and miR-302d with relative levels > 10 each.

## Discussion

Previous studies have shown that members of the miR-371 ~ 373 and miR-302/367 clusters are frequently and aberrantly expressed in extracranial GCT. Therefore, analysis of these GCT-specific miRNAs in iGCT may be an innovative and promising diagnostic tool. Here, we present the to date largest study on miRNAs as liquid biomarkers in iGCT delineating GCT-specific miRNAs in serum and CSF.

In our study miR-371a, miR-372, miR-367, miR-302a and miR-302d in serum discriminated between iGCT of different histology and controls comprising mostly healthy donors. This is in line with our (Syring et al. 2015) and others’ miRNA analyses in patients suffering from testicular GCT including a total of > 1000 serum samples (Dieckmann et al. 2019, 2012; Agthoven and Looijenga 2017). In iGCT, relative miR-371a levels in two smaller case series were similar to our study but formerly only detectable in the serum of 2/5 patients (Murray et al. 2016, 2020), maybe explained by the adaption of the miRNA quantification protocol in our study.

The analysis of a panel of miRNAs may enhance the diagnostic accuracy. Thus, in our study serum miR-302d also distinguished iGCT patients from healthy controls with a comparable expression level to miR-371a. Although miR-302d is detectable in GCT tissues of both pediatric and adult patients and is overexpressed in YST compared to germinoma (Murray et al. 2010), data on its usefulness as liquid biomarker have been lacking. Serum miR-302d has not been investigated in testicular cancer patients focusing on the miR-371 ~ 373- cluster (Gillis et al. 2013; Dieckmann et al. 2019, 2012; Agthoven and Looijenga 2017) or has been excluded from detailed analysis in extracranial GCT as its levels ranged only within two *C*_t_ values between patients and non-tumor control (Murray et al. 2016). Here we document profound expression of both miR-371a and miR-302d in all iGCT sera including serum of biomarker-negative patients. Thus, not only miR-371a but also miR-302d added value in the diagnosis of iGCT especially in biomarker-negative patients, but did not outperform miR-371a as recently been noticed in a multi-institutional pooled miRNA analysis in testicular GCT (Piao et al. 2021).

MiRNA analysis may be influenced by various pre- and post-analytical variables and normalization strategies (Myklebust et al. 2019). The latter requires an endogenous stably expressed reference gene miRNA. MiR-30b-5p fulfilled this purpose in several analyses on testicular GCT in serum (Dieckmann et al. 2019; Agthoven and Looijenga 2017; Terbuch et al. 2018; Rosas Plaza et al. 2019) and was also employed in two studies on intracranial GCT both in serum as well as in CSF (Murray et al. 2016, 2020). However, miR-30b-5p was barely detectable in another analysis in 2/4 CSF samples from healthy donors by RT-qPCR (Lobo et al. 2019). In our study in a total of 13 different CSF samples, *C*_t_ values of miR-30b-5p markedly varied within a range of twelve *C*_t_ cycles underscoring the need for a more homogeneously expressed reference gene miRNA in CSF. Previous RNAseq-based miRNA examination of 129 samples of 12 different body fluids from healthy donors identified abundant miR-204-5p expression in 11 CSF samples, thus ranking among the top five miRNAs in CSF in this study (Godoy et al. 2018). Here, we confirm the suitability of miR-204-5p for normalization purposes in miRNA analysis of the CSF by demonstrating its higher expression and lower variability compared to the broadly used reference gene miR-30b-5p.

Of note, under these optimized conditions in our study miR-371a, miR-372, miR-367 and miR-302d in CSF presented as strong indicators of iGCT disease. In addition, miR-302b was beneficial in CSF to differentiate iGCT from other diseases, such as low-grade gliomas or pituitary inflammation. In another study analyzing miRNA expression in CSF of two iGCT patients and utilizing miR-30b-5p as housekeeper, only miR-371a and miR-372 discriminated patients from those with a benign brain tumor with miR-302d being not included in the analysis (Murray et al. 2016). In addition, miR-371a analysis in CSF seems to discriminate iGCT from Langerhans cell histiocytosis (LCH) as shown in a recent case report (Murray et al. 2020). Since LCH often presents with similar radiological changes as iGCT such as thickening of the pituitary gland, miRNA analysis may add value to the routine CSF assessment in the differential diagnosis of iGCT.

While healthy donors should be preferred as comparator group for normalization, sampling of CSF remains challenging, since lumbar puncture is mainly performed in symptomatic individuals. This issue was addressed in one study by normalization of CSF-levels against Δ*C*_t_ values of serum rather than CSF controls (Murray et al. 2016). This resulted in comparatively high miR-371a expression up to $2^{{ - \Delta \Delta C_{{\text{t}}} }}$ levels of 100,000 in the CSF of the two iGCT patients (Murray et al. 2016). Similarly, normalization of CSF values in our iGCT patients against the serum Δ*C*_t_ values of our predominantly healthy controls resulted in extraordinary high relative miR-371a CSF levels (3500–470,000) in spite of raw CSF *C*_t_ values of miR-371a in our iGCT cohort left unchanged. Since miRNA distribution between CSF and serum differed in comparative microRNA analysis of healthy donors by RNAseq (Godoy et al. 2018) and qPCR (Weber et al. 2010), usage of serum Δ*C*_t_ control values for normalization of CSF miRNA data remains a matter of debate and selection of appropriate controls is critical. Five of our nine CSF control samples presented an exceedingly low maximum fluorescence in the miR-371a-qPCR although fluorescence in qPCR of the housekeeper and the other miRNAs was sufficient. The amount of miR-371a in these samples seemed to be so low that miR-371a *C*_t_ values could not be determined and the samples had to be excluded from the analysis. In the other four control samples valid *C*_t_ values could be attributed. Thus, sufficient numbers of CSF control samples for miR-371a analysis are also essential.

In our cohort of iGCT patients we documented a similar GCT-specific miRNA expression pattern between CSF and serum indicating the passage of these miRNAs across the blood–brain barrier. Similarly, in testicular cancer circulating miR-371a is specifically derived from GCT tissue and its tissue levels correlate with levels of the tumor surrounding hydrocele and levels of serum being indicative for miRNA transfer through the blood-testis barrier (Belge et al. 2020; Dieckmann et al. 2016). However, low total RNA yields and different miRNA proportions of total RNA can affect miRNA analysis in CSF. We employed a well-established, robust and quality-controlled pipeline for miRNA analysis including pre-amplification of samples (Murray et al. 2016) and increased total RNA yield by performing RNA isolation twice. Nevertheless, miR-371a analysis in CSF was impaired by low amount of miR-371a in CSF control samples and qPCR of miR-302a and miR-302c repetitively revealed non-sigmoidal but linear amplification curves in both patients and controls. As analysis of GCT-specific miRNAs in serum showed a similar pattern to miRNA assessment in CSF, serum may be preferred to CSF as it yields more RNA and can be obtained by a less invasive and easier access in both patients and healthy controls.

In iGCT, an important clinical question is the distinction between pure germinoma and NGGCT as the latter require a more intensive treatment due to inferior outcomes (Calaminus et al. 2017, 2013). Known GCT-specific miRNAs such as miR-371~ 373 do not permit discrimination between these two iGCT subtypes in our and two other recent studies (Murray et al. 2016, 2020). Thus, we analyzed miR-142-5p and miR-146a-5p in serum of iGCT patients, which were highly expressed in germinoma compared to non-germinoma tissue in a global miRNA analysis of 12 iGCT (Wang et al. 2010). Of note, as a key regulator of anti-tumor immunity, miR-142-5p inhibited PD-L1 expression on tumor cells in vitro enhancing tumor infiltration by CD4^+^- and CD8^+^-lymphocytes (Jia et al. 2017). In germinoma, detection of miR-142-5p may, therefore, reflect the immunohistochemically documented high lymphocytic infiltration in the tumor microenvironment in the absence of PD-L1 (Zapka et al. 2018). In our study overexpression of miR-142-5p and miR-146a-5p was detectable in the serum of 1/3 patients who suffered from an iGCT with partly germinoma histology. As miR-142-5p and miR-146a-5p may also be highly expressed in the peripheral blood of healthy subjects due to their regulating role in the immune response, these miRNAs may only discriminate between germinoma and NGGCT in tissue but not in serum.

Perspectively, serum miRNAs of the C19MC cluster may prove beneficial to identify NGGCT, as these miRNAs were indicative for pure embryonal carcinoma histology not only in tissue but also in the serum of patients suffering from testicular GCT (Flor et al. 2016). In a large miRNA tissue analysis of 103 testicular GCT, overexpressed miRNAs from the C19MC cluster were also indicative for NGGCT (Qin et al. 2020).

In the largest data set so far on miRNAs as liquid biomarkers in iGCT, we underlined the utility of miR-371a, miR-372, miR-367 and miR-302d in patient serum for diagnosis of iGCT, a result particularly noteworthy in germinoma patients negative for other biomarkers, such as AFP and ß-HCG. Furthermore, we confirmed the suitability of these miRNAs as liquid biomarkers in both serum and CSF of iGCT patients. Using our miRNA protocol, diagnosis of iGCT in serum samples was a feasible and valid approach, particularly as serum can be readily obtained from patients and healthy controls by a less invasive procedure. This approach complements miRNA analysis in CSF, which may be complicated due to low RNA yield. Our study has some limitations. First, only a small number of patients could be included due to the rarity of iGCT. Second, the multi-institutional approach may have resulted in different pre-analytical sample handling including different time intervals until centrifugation and cryopreservation of probes, which could have affected miRNA quality.

Verification of miR-371 ~ 373 and miR-302d for iGCT diagnosis in larger cohorts of iGCT patients and control subjects suffering from brain tumors of different histologies may facilitate the miRNA analysis into routine clinical practice with the potential to replace neurosurgical interventions by liquid biopsy. Future iGCT studies should also be geared to investigate other miRNAs in serum and/or CSF that enable for discrimination of germinoma from NGGCT. Furthermore, miRNA analysis during therapy may allow to monitor response to therapy as well as detect minimal residual disease.

## Data Availability

The data sets generated during and/or analysed during the current study are available from the corresponding author on reasonable request.

## References

[CR1] Belge G, Hennig F, Dumlupinar C, Grobelny F, Junker K, Radtke A, Dieckmann KP (2020) Graded expression of microRNA-371a-3p in tumor tissues, contralateral testes, and in serum of patients with testicular germ cell tumor. Oncotarget 11:1462–1473. 10.18632/oncotarget.27565 32363003 10.18632/oncotarget.27565 PMC7185068

[CR2] Bell E, Watson HL, Bailey S, Murray MJ, Coleman N (2017) A robust protocol to quantify circulating cancer biomarker microRNAs. Methods Mol Biol 1580:265–279. 10.1007/978-1-4939-6866-4_18 28439839 10.1007/978-1-4939-6866-4_18

[CR3] Calaminus G, Bamberg M, Baranzelli MC, Benoit Y, di Montezemolo LC, Fossati-Bellani F, Jurgens H, Kuhl HJ, Lenard HG, Curto ML et al (1994) Intracranial germ cell tumors: a comprehensive update of the European data. Neuropediatrics 25:26–32. 10.1055/s-2008-1071577 8208347 10.1055/s-2008-1071577

[CR4] Calaminus G, Andreussi L, Garre ML, Kortmann RD, Schober R, Göbel U (1997) Secreting germ cell tumors of the central nervous system (CNS). First results of the cooperative German/Italian pilot study (CNS sGCT). Klin Padiatr 209:222–227. 10.1055/s-2008-1043954 9293454 10.1055/s-2008-1043954

[CR5] Calaminus G, Kortmann R, Worch J, Nicholson JC, Alapetite C, Garre ML, Patte C, Ricardi U, Saran F, Frappaz D (2013) SIOP CNS GCT 96: final report of outcome of a prospective, multinational nonrandomized trial for children and adults with intracranial germinoma, comparing craniospinal irradiation alone with chemotherapy followed by focal primary site irradiation for patients with localized disease. Neuro Oncol 15:788–796. 10.1093/neuonc/not019 23460321 10.1093/neuonc/not019 PMC3661100

[CR6] Calaminus G, Frappaz D, Kortmann RD, Krefeld B, Saran F, Pietsch T, Vasiljevic A, Garre ML, Ricardi U, Mann JR et al (2017) Outcome of patients with intracranial non-germinomatous germ cell tumors-lessons from the SIOP-CNS-GCT-96 trial. Neuro Oncol 19:1661–1672. 10.1093/neuonc/nox122 29048505 10.1093/neuonc/nox122 PMC5716085

[CR7] Dieckmann KP, Spiekermann M, Balks T, Flor I, Loning T, Bullerdiek J, Belge G (2012) MicroRNAs miR-371-3 in serum as diagnostic tools in the management of testicular germ cell tumours. Br J Cancer 107:1754–1760. 10.1038/bjc.2012.469 23059743 10.1038/bjc.2012.469 PMC3493876

[CR8] Dieckmann KP, Spiekermann M, Balks T, Ikogho R, Anheuser P, Wosniok W, Loening T, Bullerdiek J, Belge G (2016) MicroRNA miR-371a-3p - a novel serum biomarker of testicular germ cell tumors: evidence for specificity from measurements in testicular vein blood and in neoplastic hydrocele fluid. Urol Int 97:76–83. 10.1159/000444303 26989896 10.1159/000444303

[CR9] Dieckmann KP, Radtke A, Geczi L, Matthies C, Anheuser P, Eckardt U, Sommer J, Zengerling F, Trenti E, Pichler R et al (2019) Serum levels of microRNA-371a-3p (M371 test) as a new biomarker of testicular germ cell tumors: results of a prospective multicentric study. J Clin Oncol 37:1412–1423. 10.1200/JCO.18.01480 30875280 10.1200/JCO.18.01480 PMC6544462

[CR10] Flor I, Spiekermann M, Loning T, Dieckmann KP, Belge G, Bullerdiek J (2016) Expression of microRNAs of C19MC in different histological types of testicular germ cell tumour. Cancer Genomics Proteomics 13:281–289 27365378

[CR11] Gillis AJ, Stoop HJ, Hersmus R, Oosterhuis JW, Sun Y, Chen C, Guenther S, Sherlock J, Veltman I, Baeten J et al (2007) High-throughput microRNAome analysis in human germ cell tumours. J Pathol 213:319–328. 10.1002/path.2230 17893849 10.1002/path.2230

[CR12] Gillis AJ, Rijlaarsdam MA, Eini R, Dorssers LC, Biermann K, Murray MJ, Nicholson JC, Coleman N, Dieckmann KP, Belge G et al (2013) Targeted serum miRNA (TSmiR) test for diagnosis and follow-up of (testicular) germ cell cancer patients: a proof of principle. Mol Oncol 7:1083–1092. 10.1016/j.molonc.2013.08.002 24012110 10.1016/j.molonc.2013.08.002 PMC5528443

[CR13] Godoy PM, Bhakta NR, Barczak AJ, Cakmak H, Fisher S, MacKenzie TC, Patel T, Price RW, Smith JF, Woodruff PG et al (2018) Large differences in small RNA composition between human biofluids. Cell Rep 25:1346–1358. 10.1016/j.celrep.2018.10.014 30380423 10.1016/j.celrep.2018.10.014 PMC6261476

[CR14] Ichimura K, Fukushima S, Totoki Y, Matsushita Y, Otsuka A, Tomiyama A, Niwa T, Takami H, Nakamura T, Suzuki T et al (2016) Recurrent neomorphic mutations of MTOR in central nervous system and testicular germ cell tumors may be targeted for therapy. Acta Neuropathol 131:889–901. 10.1007/s00401-016-1557-x 26956871 10.1007/s00401-016-1557-x

[CR15] Jia L, Xi Q, Wang H, Zhang Z, Liu H, Cheng Y, Guo X, Zhang J, Zhang Q, Zhang L et al (2017) miR-142-5p regulates tumor cell PD-L1 expression and enhances anti-tumor immunity. Biochem Biophys Res Commun 488:425–431. 10.1016/j.bbrc.2017.05.074 28511795 10.1016/j.bbrc.2017.05.074

[CR16] Lobo J, Gillis AJM, van den Berg A, Dorssers LCJ, Belge G, Dieckmann KP, Roest HP, van der Laan LJW, Gietema J, Hamilton RJ et al (2019) Identification and validation model for informative liquid biopsy-based microRNA biomarkers: insights from germ cell tumor in vitro, in vivo and patient-derived data. Cells. 10.3390/cells8121637 31847394 10.3390/cells8121637 PMC6952794

[CR17] Murray M, Schönberger S (2014) Biology of germ cell tumors. In: Frazier A, Amatruda J (eds) Pediatric germ cell tumors. Springer, Berlin, Heidelberg, Heidelberg, pp 1–15

[CR18] Murray MJ, Saini HK, van Dongen S, Palmer RD, Muralidhar B, Pett MR, Piipari M, Thornton CM, Nicholson JC, Enright AJ et al (2010) The two most common histological subtypes of malignant germ cell tumour are distinguished by global microRNA profiles, associated with differential transcription factor expression. Mol Cancer 9:290. 10.1186/1476-4598-9-290 21059207 10.1186/1476-4598-9-290 PMC2993676

[CR19] Murray MJ, Bell E, Raby KL, Rijlaarsdam MA, Gillis AJ, Looijenga LH, Brown H, Destenaves B, Nicholson JC, Coleman N (2016) A pipeline to quantify serum and cerebrospinal fluid microRNAs for diagnosis and detection of relapse in paediatric malignant germ-cell tumours. Br J Cancer 114:151–162. 10.1038/bjc.2015.429 26671749 10.1038/bjc.2015.429 PMC4815809

[CR20] Murray MJ, Ajithkumar T, Harris F, Williams RM, Jalloh I, Cross J, Ronghe M, Ward D, Scarpini CG, Nicholson JC et al (2020) Clinical utility of circulating miR-371a-3p for the management of patients with intracranial malignant germ cell tumors. Neurooncol Adv 2:vdaa048. 10.1093/noajnl/vdaa048 32642701 10.1093/noajnl/vdaa048 PMC7236383

[CR21] Myklebust MP, Rosenlund B, Gjengsto P, Bercea BS, Karlsdottir A, Brydoy M, Dahl O (2019) Quantitative PCR measurement of miR-371a-3p and miR-372-p is influenced by hemolysis. Front Genet 10:463. 10.3389/fgene.2019.00463 31191602 10.3389/fgene.2019.00463 PMC6539204

[CR22] Palmer RD, Murray MJ, Saini HK, van Dongen S, Abreu-Goodger C, Muralidhar B, Pett MR, Thornton CM, Nicholson JC, Enright AJ et al (2010) Malignant germ cell tumors display common microRNA profiles resulting in global changes in expression of messenger RNA targets. Can Res 70:2911–2923. 10.1158/0008-5472.CAN-09-3301 10.1158/0008-5472.CAN-09-3301 PMC3000593 20332240

[CR23] Piao J, Lafin JT, Scarpini CG, Nuño MM, Syring I, Dieckmann K-P, Belge G, Ellinger J, Amatruda JF, Bagrodia A et al (2021) A multi-institutional pooled analysis demonstrates that circulating miR-371a-3p alone is sufficient for testicular malignant germ cell tumor diagnosis. Clin Genitourin Cancer 19:469–479. 10.1016/j.clgc.2021.08.006 34629299 10.1016/j.clgc.2021.08.006 PMC9084514

[CR24] Qin G, Mallik S, Mitra R, Li A, Jia P, Eischen CM, Zhao Z (2020) MicroRNA and transcription factor co-regulatory networks and subtype classification of seminoma and non-seminoma in testicular germ cell tumors. Sci Rep 10:852. 10.1038/s41598-020-57834-w 31965022 10.1038/s41598-020-57834-w PMC6972857

[CR25] Rosas Plaza X, van Agthoven T, Meijer C, van Vugt M, de Jong S, Gietema JA, Looijenga LHJ (2019) miR-371a-3p, miR-373-3p and miR-367-3p as serum biomarkers in metastatic testicular germ cell cancers before, during and after chemotherapy. Cells. 10.3390/cells8101221 31597402 10.3390/cells8101221 PMC6830325

[CR26] Schneider DT, Zahn S, Sievers S, Alemazkour K, Reifenberger G, Wiestler OD, Calaminus G, Göbel U, Perlman EJ (2006) Molecular genetic analysis of central nervous system germ cell tumors with comparative genomic hybridization. Mod Pathol 19:864–873. 10.1038/modpathol.3800607 16607373 10.1038/modpathol.3800607

[CR27] Schulz M, Afshar-Bakshloo M, Koch A, Capper D, Driever PH, Tietze A, Grun A, Thomale UW (2021) Management of pineal region tumors in a pediatric case series. Neurosurg Rev 44:1417–1427. 10.1007/s10143-020-01323-1 32504201 10.1007/s10143-020-01323-1 PMC8121748

[CR28] Syring I, Bartels J, Holdenrieder S, Kristiansen G, Muller SC, Ellinger J (2015) Circulating serum miRNA (miR-367-3p, miR-371a-3p, miR-372-3p and miR-373-3p) as biomarkers in patients with testicular germ cell cancer. J Urol 193:331–337. 10.1016/j.juro.2014.07.010 25046619 10.1016/j.juro.2014.07.010

[CR29] Takami H, Fukuoka K, Fukushima S, Nakamura T, Mukasa A, Saito N, Yanagisawa T, Nakamura H, Sugiyama K, Kanamori M et al (2019) Integrated clinical, histopathological, and molecular data analysis of 190 central nervous system germ cell tumors from the iGCT Consortium. Neuro Oncol 21:1565–1577. 10.1093/neuonc/noz139 31420671 10.1093/neuonc/noz139 PMC6917411

[CR30] Terbuch A, Adiprasito JB, Stiegelbauer V, Seles M, Klec C, Pichler GP, Resel M, Posch F, Lembeck AL, Stoger H et al (2018) MiR-371a-3p serum levels are increased in recurrence of testicular germ cell tumor patients. Int J Mol Sci. 10.3390/ijms19103130 30321995 10.3390/ijms19103130 PMC6213366

[CR31] van Agthoven T, Looijenga LHJ (2017) Accurate primary germ cell cancer diagnosis using serum based microRNA detection (ampTSmiR test). Oncotarget 8:58037–58049. 10.18632/oncotarget.10867 28938535 10.18632/oncotarget.10867 PMC5601631

[CR32] Wang HW, Wu YH, Hsieh JY, Liang ML, Chao ME, Liu DJ, Hsu MT, Wong TT (2010) Pediatric primary central nervous system germ cell tumors of different prognosis groups show characteristic miRNome traits and chromosome copy number variations. BMC Genomics 11:132. 10.1186/1471-2164-11-132 20178649 10.1186/1471-2164-11-132 PMC2837036

[CR33] Weber JA, Baxter DH, Zhang S, Huang DY, Huang KH, Lee MJ, Galas DJ, Wang K (2010) The microRNA spectrum in 12 body fluids. Clin Chem 56:1733–1741. 10.1373/clinchem.2010.147405 20847327 10.1373/clinchem.2010.147405 PMC4846276

[CR34] Zapka P, Dörner E, Dreschmann V, Sakamato N, Kristiansen G, Calaminus G, Vokuhl C, Leuschner I, Pietsch T (2018) Type, frequency, and spatial distribution of immune cell infiltrates in CNS germinomas: evidence for inflammatory and immunosuppressive mechanisms. J Neuropathol Exp Neurol 77:119–127. 10.1093/jnen/nlx106 29237087 10.1093/jnen/nlx106

